# Unravelling the molecular mechanisms causal to type 2 diabetes across global populations and disease-relevant tissues

**DOI:** 10.1038/s42255-025-01444-1

**Published:** 2026-01-27

**Authors:** Ozvan Bocher, Ana Luiza Arruda, Satoshi Yoshiji, Chi Zhao, Alicia Huerta-Chagoya, Chen-Yang Su, Xianyong Yin, Davis Cammann, Henry J. Taylor, Jingchun Chen, Ken Suzuki, Ravi Mandla, Ta-Yu Yang, Fumihiko Matsuda, Josep M. Mercader, Jason Flannick, James B. Meigs, Alexis C. Wood, Marijana Vujkovic, Benjamin F. Voight, Cassandra N. Spracklen, Jerome I. Rotter, Andrew P. Morris, Eleftheria Zeggini

**Affiliations:** 1 Institute of Translational Genomics, Helmholtz Zentrum München—German Research Center for Environmental Health, Neuherberg, Germany; 2 Univ Brest, Inserm, EFS, UMR 1078, GGB, Brest, France; 3 Technical University of Munich (TUM), School of Medicine and Health, Graduate School of Experimental Medicine, Munich, Germany; 4 Medical Research Council (MRC) Epidemiology Unit, University of Cambridge, Cambridge, UK; 5 Programs in Metabolism and Medical and Population Genetics, Broad Institute of MIT and Harvard, Cambridge, MA USA; 6 Center for Genomic Medicine, Kyoto University Graduate School of Medicine, Kyoto, Japan; 7 McGill Genome Centre, McGill University, Montreal, Quebec Canada; 8 Department of Biostatistics and Epidemiology, University of Massachusetts Amherst, Amherst, MA USA; 9 Diabetes Unit and Center for Genomic Medicine, Massachusetts General Hospital, Boston, MA USA; 10 Harvard Medical School, Boston, MA USA; 11 Canada Excellence Research Chair in Genomic Medicine, McGill University, Montréal, Québec Canada; 12 Quantitative Life Sciences, McGill University, Montreal, Québec Canada; 13 Department of Epidemiology, School of Public Health, Nanjing Medical University, Nanjing, China; 14 Department of Biostatistics and Center for Statistical Genetics, University of Michigan, Ann Arbor, MI USA; 15 Nevada Institute of Personalized Medicine, University of Nevada, Las Vegas, Las Vegas, NV USA; 16 Center for Precision Health Research, National Human Genome Research Institute, National Institutes of Health, Bethesda, MD USA; 17 British Heart Foundation Cardiovascular Epidemiology Unit, Department of Public Health and Primary Care, University of Cambridge, Cambridge, UK; 18 Heart and Lung Research Institute, University of Cambridge, Cambridge, UK; 19 Department of Diabetes and Metabolic Diseases, Graduate School of Medicine, University of Tokyo, Tokyo, Japan; 20 Division of Genetics and Genomics, Boston Children’s Hospital, Boston, MA USA; 21 Department of Pediatrics, Boston Children’s Hospital, Boston, MA USA; 22 Department of Medicine, Harvard Medical School, Boston, MA USA; 23 Division of General Internal Medicine, Massachusetts General Hospital, Boston, MA USA; 24 USDA/ARS Children’s Nutrition Research Center, Baylor College of Medicine, Houston, TX USA; 25 Corporal Michael J. Crescenz VA Medical Center, Philadelphia, PA USA; 26 Department of Genetics, University of Pennsylvania Perelman School of Medicine, Philadelphia, PA USA; 27 Department of Biostatistics, Epidemiology and Informatics, University of Pennsylvania Perelman School of Medicine, Philadelphia, PA USA; 28 Department of Systems Pharmacology and Translational Therapeutics, University of Pennsylvania Perelman School of Medicine, Philadelphia, PA USA; 29 Institute for Translational Medicine and Therapeutics, University of Pennsylvania Perelman School of Medicine, Philadelphia, PA USA; 30 Institute for Translational Genomics and Population Sciences, Department of Pediatrics, Lundquist Institute for Biomedical Innovation at Harbor—UCLA Medical Center, Torrance, CA USA; 31 Centre for Genetics and Genomics Versus Arthritis, Centre for Musculoskeletal Research, The University of Manchester, Manchester, UK; 32 NIHR Manchester Biomedical Research Centre, Manchester University NHS Foundation Trust, Manchester, UK; 33 Technical University of Munich (TUM), TUM University Hospital, TUM School of Medicine and Health, Munich, Germany

**Keywords:** Type 2 diabetes, Statistical methods, Metabolism, Genome-wide association studies, Genetics

## Abstract

Type 2 diabetes (T2D) is a prevalent disease arising from complex molecular mechanisms. Here we leverage T2D genetic associations to identify causal molecular mechanisms in an ancestry-aware and tissue-aware manner. Using two-sample Mendelian randomization corroborated by colocalization across four global ancestries, we analyse 20,307 gene and 1,630 protein expression levels using blood-derived *cis*-quantitative trait loci (QTLs). We detect causal effects of genetically predicted levels of 335 genes and 46 proteins on T2D risk, with 16.4% and 50% replication in independent cohorts, respectively. Using gene expression *cis*-QTLs derived from seven T2D-relevant tissues, we identify causal links between the expression of 676 genes and T2D risk, refining known associations such as *BAK1* and describing additional ones like *CPXM1*. Causal effects are mostly shared across ancestries but are highly heterogeneous across tissues. Our findings provide insights into cross-ancestry and tissue-informed multi-omics causal inference approaches and demonstrate their power in uncovering molecular processes driving T2D.

## Abstract (web summary)

Analysing the causal links of gene expression and protein abundance on type 2 diabetes risk in blood and seven tissues related to the disease from individuals of four ancestries, the authors advance our understanding of the genetic architecture of type 2 diabetes

## Main

T2D is a prevalent, complex disease. Large-scale genome-wide association study (GWAS) meta-analyses^1,2^ have advanced the understanding of its underlying genetic architecture. As T2D is a heterogeneous disease^3^ with different underlying pathologies among individuals, identifying different causal biological mechanisms can facilitate potential leads for drug development^4^ and interventions in patient populations that maximize clinical benefit. Assessing whether these mechanisms are causal remains challenging but can be inferred from dedicated statistical methods. One such method is Mendelian randomization (MR), which relies on the same principle as randomized clinical trials, in which genetic variants that predict the exposure of interest are used. As these variants are randomly assigned at conception, they are generally not influenced by lifestyle or environmental factors later in life, reducing the risk of reverse causation and confounding. In MR, these genetic variants are then used as instrumental variables (IVs)^5^ to predict the exposure and assess its putative causal effect on the outcome, provided specific assumptions are met. Using quantitative trait loci (QTLs) as IVs for molecular phenotypes, MR has been successfully used to provide evidence of causal effects of methylation^6,7^, gene expression^8^ and protein^9^ or metabolite levels^10,11^ on T2D risk. However, most of these studies have been performed using blood levels of the putative causal risk factors and have not addressed mechanisms in disease-relevant tissues underlying genetic associations^12^. Given that gene expression and its genetic regulation vary across tissues^13,14^, previous causal relationships investigated in blood might not represent biological mechanisms directly linked to the disease and might miss causal mechanisms relevant to complex diseases^15^. For example, the authors of the S-PrediXcan method highlighted that most associations between GWAS phenotypes and predicted gene expression were tissue-specific^16^. At the protein level, blood is predicted to be more informative, and protein QTLs (pQTLs) have been shown to be more enriched for trait-causal variants than gene expression QTLs (eQTLs) in this tissue^17^. Additionally, the majority of causal inference studies have focused on populations that are genetically similar to Europeans (EUR; based on the 1000 Genomes Project phase 3 (ref. ^18^) individuals sampled from continental Europe as a reference). However, prevalence and manifestations of complex traits such as T2D are known to vary across populations^19^, which might, at least partly, be explained by differences in expression levels driven by genetic stratification^20^. Investigating only EUR, therefore, limits the inference of causal molecular mechanisms across diverse global populations. Given the heterogeneity in QTL mapping across populations described in previous studies, assessing QTLs in diverse groups facilitates the assessment of molecular traits for which IVs are not available in EUR^21^. However, even when available in non-EUR, QTL studies are limited in sample size and mostly restricted to blood levels^22^.

To expand diversity in T2D genetic studies and improve the generalizability of findings, the T2D Global Genomics Initiative (T2DGGI) has performed a multi-ancestry GWAS meta-analysis, gathering genome-wide data from over 2.5 million individuals, including over 700,000 individuals of non-European ancestry^1^. A previous study^23^ integrated these T2DGGI data with large-scale omics, identifying evidence of shared signals (that is, colocalization^24^) between molecular traits and 56% of the 1,289 T2DGGI GWAS meta-analysis index variants, highlighting the benefit of interrogating data from under-represented global populations. Here, we study the causal links of gene expression and protein abundance levels with T2D risk using MR approaches based on *cis-*QTLs, which are predicted to have the strongest biological impact on molecular traits^25^. We expand previous causal inference studies by leveraging the largest set of T2D genetic associations reported by T2DGGI and blood QTL maps from global populations in single-ancestry analyses and multi-ancestry meta-analyses and investigating causal effects in seven further tissues relevant to T2D. To corroborate our findings, we perform colocalization analysis using a method suitable for polygenic traits, such as T2D, as it allows for multiple causal variants at each interrogated genetic locus.

## Results

MR analyses were performed using QTLs as IVs. Throughout this paper, we refer to ‘genes’ for gene expression levels genetically predicted from *cis-*eQTLs and to ‘proteins’ for protein levels genetically predicted from *cis-*pQTLs. We use ‘molecular traits’ to refer to genes and proteins identified in any MR analysis.

### Study design overview

**Fig. 1:**
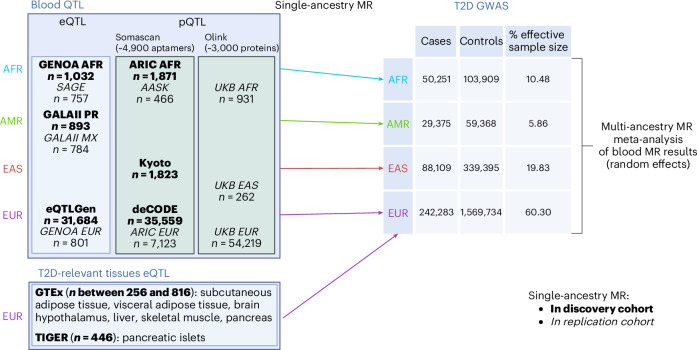
Overview of the cohorts and tissues used to perform single-ancestry MR analyses in populations genetically similar to EUR, AFR, AMR and EAS based on the 1000 Genomes Project phase 3 (ref. ^18^). Discovery cohorts are indicated in bold, and replication cohorts for blood MR analyses are in italics. Created in BioRender.com.

We performed blood MR analyses by using IVs defined from blood eQTLs and plasma pQTLs from multiple cohorts (Fig. 1 and Supplementary Table 1). Analyses were conducted in an ancestry-aware manner; that is, within ancestry groups genetically similar to EUR^26,27^, Africans (AFR^28,29^), admixed Americans (AMR^30^) and East-Asians (EAS^31^) (Fig. 1). Causal effects were considered based on several lines of evidence coming from statistical significance corrected for multiple testing, sensitivity analysis and colocalization (STROBE guidelines; see Methods). Replication was performed in matched genetic ancestry groups^28–30,32,33^, depending on data availability (Fig. 1). We meta-analysed MR results across genetic ancestry groups, where possible, for either genes or proteins, separately (Methods). We also performed *cis-*eQTL MR analysis in seven additional T2D-relevant tissues^34,35^ (whole pancreas, pancreatic islets, brain hypothalamus, visceral adipose, subcutaneous adipose, liver and skeletal muscle). We report causal estimates as odds ratios (ORs) per unit of genetically predicted gene expression and protein levels on T2D risk. We refer to protective effects of molecular traits for which the OR is lower than 1; that is, for which increased levels of expression are associated with reduced T2D risk, and to deleterious effects otherwise. Although blood eQTL MR is not the most tissue-relevant for T2D, we performed this analysis to allow comparison between global populations, given that non-EUR eQTL data from other tissues are not currently available.

### Assessing QTLs in global populations improves the detection of causal effects

**Fig. 2:**
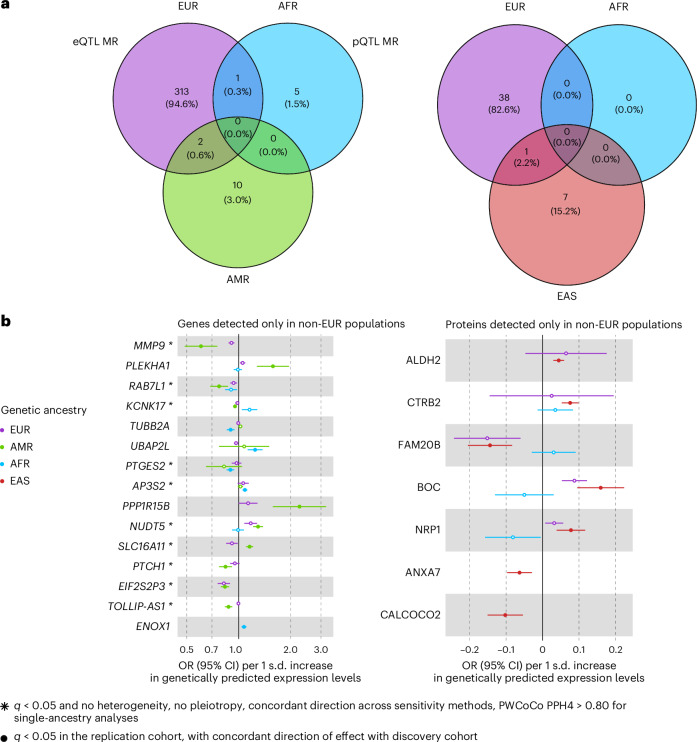
Molecular traits with causal effects identified in the blood eQTL and pQTL MR analyses. **a**, Venn diagrams representing the number of genes and proteins with causal effects identified in the three ancestry groups. **b**, Forest plots of the causal effects identified only in non-EUR from the blood eQTL and pQTL MR analyses. Causal estimates from the single-ancestry MR in the discovery cohorts (matched genetic ancestry group between the exposure and the outcome) are represented. Filled dots represent causal estimates from MR analyses that have an FDR-corrected *p* value (*q* value) of <0.05 and pass the sensitivity criteria and show evidence of colocalization (PPH4 > 0.8) in single-ancestry analyses. Genes and proteins with causal effects identified in single-ancestry analyses and replicated in independent cohorts from the same genetic ancestry group are denoted with a star. Points represent MR causal estimates derived from summary statistics (OR for T2D per 1 s.d. change in genetically predicted gene expression or protein levels, measure of centre) and error bars represent 95% confidence interval (CI). Sample sizes of the QTL datasets used as exposure datasets: (1) eQTL EUR (*n* = 31,684), AFR (*n* = 1,032), AMR (*n* = 893); (2) pQTL EUR (*n* = 35,559), AFR (*n* = 1,871), EAS (*n* = 1,823). Sample size of the T2D GWAS meta-analysis used as outcome datasets: EUR (*n* = 242,283 cases and *n* = 1,569,734 controls), AFR (*n* = 50,251 cases and *n* = 103,909 controls), AMR (*n* = 29,375 cases and *n* = 59,368 controls), EAS (*n* = 88,109 cases and *n* = 339,395 controls). Source data

**Extended Data Fig. 1:**
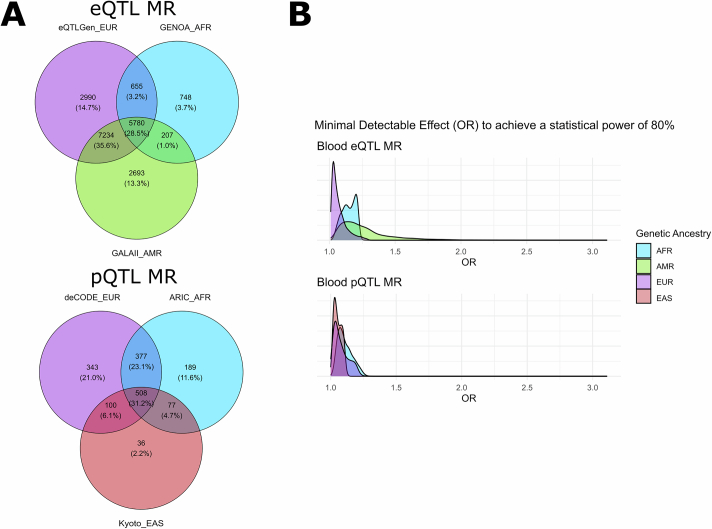
Statistical power of the causal inference analysis across ancestries. (**A**) Venn-diagrams representing the overlap of molecular traits tested in the different genetic ancestry groups (EUR, AFR and AMR for the eQTL MR analysis; EUR, AFR and EAS for the pQTL MR analysis). **(B)** Distribution of the minimal OR detectable at a statistical power of 80% in the different genetic ancestry groups for the blood eQTL and the blood pQTL MR analyses. Source data

**Extended Data Fig. 2:**
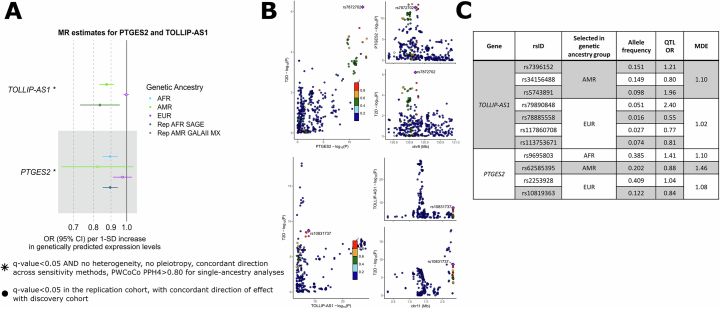
Causal estimates and frequency and effect sizes of IVs on *TOLLIP-AS1* and *PTGES2.* (**A**) Forest plots of causal estimates for *TOLLIP-AS1* and *PTGES2* from the eQTL MR analyses. Causal estimates from the single-ancestry MR in the discovery cohorts (matched genetic ancestry group between the exposure and the outcome) are also represented. Filled dots represent causal estimates from MR analyses that have a q-value < 0.05, and pass the sensitivity criteria and show evidence of colocalization (PPH4 > 0.8) in single-ancestry analyses. Genes and proteins with causal effects identified in single-ancestry analyses and replicated in independent cohorts from the same genetic ancestry group are denoted with a star. Points represent MR causal estimates derived from summary statistics (OR for T2D per SD change in genetically predicted gene expression or protein levels, measure of center) and error bars represent 95% CI. Sample sizes of the eQTL datasets used as exposure datasets: EUR (discovery n = 31,684, replication n = 801), AFR (discovery n = 1,032, replication n = 757), AMR (discovery n = 893, replication n = 784). Sample size of the T2D GWAS meta-analysis used as outcome datasets: EUR (n = 242,283 cases and n = 1,569734 controls), AFR (n = 50,251 cases and n = 103,909 controls), AMR (n = 29,375 cases and n = 59,368 controls). **(B)** LocusCompare and LocusZoom for the two genes demonstrating colocalization evidence. **(C)** Allele frequency of the IVs selected for the two genes, their association with the corresponding gene expression levels, and the MDE to achieve a statistical power of 80%. Abbreviations: QTL=Quantitative trait loci; OR=Odds ratio; SD=Standard deviation; CI=Confidence Interval; PPH4=Posterior probability of hypothesis four; MDE: Minimal detectable effect. Source data

We defined IVs in blood for a total of 20,307 genes and 1,630 proteins in at least one genetic ancestry group (Extended Data Fig. 1a). Causal effects of 331 genes were detected in at least one single-ancestry MR analysis, of which 316 (95.5%) were found in the EUR analysis (Fig. 2). This high proportion reflects the higher expected statistical power in EUR owing to the higher sample size of both the exposure and outcome datasets (Methods and Extended Data Fig. 1b). Yet a total of 15 genes showed significant causal effects only in non-EUR (ten in AMR and five in AFR). Of these 15 genes, nine (60%) were replicated in independent cohorts from matched genetic ancestry groups, strengthening the evidence for their potential causal role in T2D risk (Fig. 2a and Supplementary Tables 4 and 5). Two examples include *TOLLIP-AS1*, detected in AMR (OR = 0.87, *q* = 2.25 × 10^−4^) and *PTGES2*, detected in AFR (OR = 0.90, *q* = 2.40 × 10^−2^), with protective effects against T2D risk (Extended Data Fig. 2a,b). When investigating the allele frequencies and effect sizes of the IVs of these two genes, we observed different patterns across genetic ancestry groups. The allele frequencies of the IVs used for *PTGES2* across AFR and EUR were similar, while the effect sizes on gene expression were larger in AFR than in EUR. Conversely, for *TOLLIP-AS1*, the effect sizes on gene expression were of the same magnitude among AMR and EUR, but the allele frequencies of the IVs were higher in AMR than in EUR (Extended Data Fig. 2c). For these two genes, the expected statistical power was the highest in EUR, probably because of the higher sample size of the outcome GWAS in this genetic ancestry group. Despite having the highest statistical power, we were not able to detect causal effects of these two genes in EUR. More comprehensive and diverse data will be needed to claim potential ancestry-specific causal effects.

**Extended Data Fig. 3:**
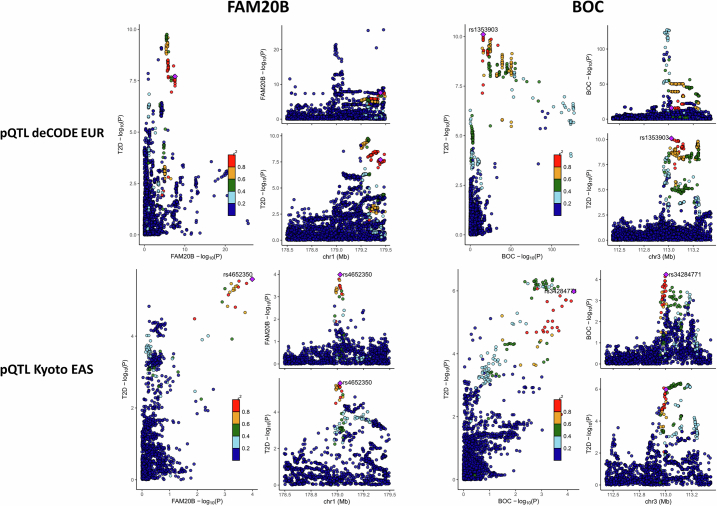
LocusCompare and LocusZoom for FAM20B and BOC, two proteins with significant causal effects detected only in EAS. Results are displayed in EUR (top panel) and in EAS (bottom panel).

Similarly, there were 46 proteins with causal effects detected in single-ancestry MR analyses, of which 39 (85%) were detected in EUR. The remaining seven proteins with causal effects detected only in non-EUR were detected in EAS (Fig. 2b). Two of them were tested only in EAS (ANXA7 and CALCOCO2), while the remaining five were tested in multiple ancestries but were significant only in EAS: CTRB2 (OR = 1.08, *q* = 3.60 × 10^−8^), FAM20B (OR = 0.87, *q* = 3.80 × 10^−4^), BOC (OR = 1.17, *q* = 2.17 × 10^−4^), NRP1 (OR = 1.08, *q* = 6.59 × 10^−3^) and ALDH2 (OR = 1.05, *q* = 6.70 × 10^−7^). Several of these proteins presented causal estimates in EUR close to the estimates in EAS, while not being considered causal in EUR. This is the case for BOC and FAM20B, which presented significant causal estimates in the EUR MR analyses but did not show evidence of colocalization in this ancestry group (BOC: posterior probability of hypothesis four (PPH4) = 0.068 in EUR vs PPH4 = 0.919 in EAS; FAM20B: PPH4 = 3.2 × 10^−5^ in EUR vs PPH4 = 0.974 in EAS). The EUR pQTL dataset used in our study comes from an Icelandic population dataset (deCODE). The lack of colocalization in EUR for these proteins may be the result of a linkage disequilibrium mismatch between Icelandic pQTL data and the EUR GWAS meta-analysis from T2DGGI. For EAS, colocalization was identified possibly owing to a more similar linkage disequilibrium pattern between the pQTL and EAS GWAS meta-analysis from T2DGGI (Extended Data Fig. 3). None of the proteins with causal effects identified in EAS could be tested for replication in this genetic ancestry group because of the lack of a replication cohort with Somascan proteomics data and the lack of IVs in the EAS UK Biobank^36^ proteomics data (Supplementary Table 6).

**Fig. 3:**
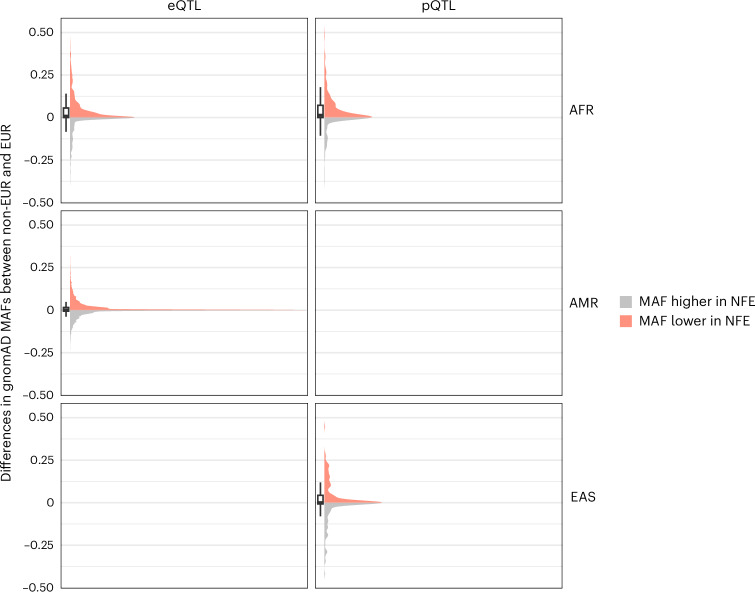
Distribution of differences between EUR and non-EUR in the minor allele frequency of IVs for genes and proteins only tested in non-EUR. The differences were computed as ${\mathrm{MAF}}_{\mathrm{non}-\mathrm{EUR}}-{\mathrm{MAF}}_{\mathrm{EUR}}$. Minor allele frequencies (MAFs) were obtained from the Genome Aggregation Database (gnomAD)^78^. gnomADg_NFE_MAF refers to the MAF observed in gnomAD genomes in the non-Finnish European (NFE) population. Positive differences—that is, IVs for which the MAF is lower in NFE than in the corresponding non-EUR ancestry group—are represented in red, and negative differences in grey. Boxplots represent the median (centre line) and the 25th and 75th percentiles (box bounds), with whiskers extending to 1.5× the interquartile range (IQR) from the box. Number of IVs included genes only tested in AFR (*n* = 1,539 IVs) and AMR (*n* = 7,262 IVs), and proteins only tested in AFR (*n* = 462 IVs) and EAS (*n* = 231 IVs). Source data

Our results further reveal the benefits of investigating non-EUR QTLs, given the increased number of genes and proteins for which IVs are available only in non-EUR. Here, a total of 6,431 genes and 570 proteins were tested only in one genetic ancestry group, of which 3,648 genes (56.7%) and 302 proteins (53.0%) were only tested in non-EUR (Extended Data Fig. 1a). Most of these IVs show minor allele frequencies lower in EUR than in non-EUR, especially when considering IVs of genes and proteins tested only in AFR (Fig. 3). It would therefore require larger sample sizes in EUR QTL studies to detect these IVs, given the expected homogeneous effect sizes across genetic ancestry groups^37^. Examples include *ENOX1* (OR = 1.07, *q* = 3.80 × 10^−2^ in AFR eQTL MR), ANXA7 (OR = 0.94, *q* = 1.16 × 10^−2^ in EAS pQTL MR) and CALCOCO2 (OR = 0.90, *q* = 3.61 × 10^−3^ in EAS pQTL MR). *CALCOCO2* is a gene previously suggested to be associated with T2D^38^, with a knockdown decreasing insulin content in the human pancreatic beta cell line EndoC-βH1 (ref. ^39^). Here, we corroborate this finding with evidence of a protective effect of increased blood expression levels of CALCOCO2 against T2D risk.

### Low ancestry-related heterogeneity for blood molecular traits causal to T2D risk

**Fig. 4:**
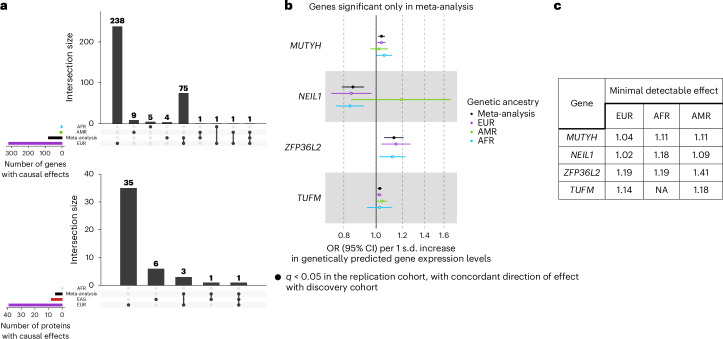
Molecular traits with causal effects identified in the blood eQTL and pQTL MR meta-analyses across ancestries. **a**, Upset plots representing genes and proteins with causal effects identified in the three ancestry groups and in the cross-ancestry meta-analysis. **b**, Forest plots of the causal effects identified only in the blood eQTL MR meta-analysis. Filled dots represent causal estimates from MR analyses that have *q* < 0.05 and (1) pass the sensitivity criteria and show evidence of colocalization (PPH4 > 0.8) in single-ancestry analyses or (2) present nominal significance and meet criterion 1 in at least one cohort entering the meta-analysis. Points represent MR causal estimates derived from summary statistics (OR for T2D per 1 s.d. change in genetically predicted gene expression or protein levels, measure of centre); error bars, 95% CI. Sample sizes of the QTL datasets used as exposure datasets: (1) eQTL EUR (*n* = 31,684), AFR (*n* = 1,032), AMR (*n* = 893); (2) pQTL EUR (*n* = 35,559), AFR (*n* = 1,871), EAS (*n* = 1,823). Sample size of the T2D GWAS meta-analysis used as outcome datasets: EUR (*n* = 242,283 cases and *n* = 1,569,734 controls), AFR (*n* = 50,251 cases and *n* = 103,909 controls), AMR (*n* = 29,375 cases and *n* = 59,368 controls), EAS (*n* = 88,109 cases and *n* = 339,395 controls). **c**, Minimal detectable effect (OR) to achieve a statistical power of 80% in the three genetic ancestry groups for the four genes identified only in the eQTL meta-analysis. Source data

**Extended Data Fig. 4:**
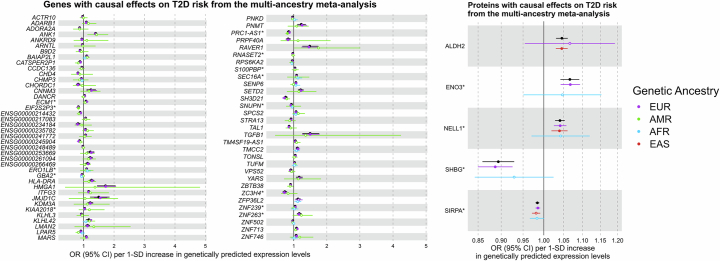
Genes and proteins with causal effects identified in the MR multi-ancestry meta-analysis. Causal estimates from the single-ancestry MR in the discovery cohorts are also depicted. Filled dots represent causal estimates from MR analyses that have a q-value < 0.05, and (1) pass the sensitivity criteria and show evidence of colocalization (PPH4 > 0.8) in single-ancestry analyses, or (2) present nominal significance and meet criteria (1) in at least one cohort entering the meta-analysis. Genes and proteins with causal effects identified in single-ancestry analyses and replicated in independent cohorts from the same genetic ancestry group are denoted with a star. Points represent MR causal estimates derived from summary statistics (OR for T2D per SD change in genetically predicted gene expression or protein levels, measure of center) and error bars represent 95% CI. Sample sizes of the QTL datasets used as exposure datasets: **(1) eQTL** EUR (n = 31,684), AFR (n = 1,032), AMR (n = 893) **(2) pQTL** EUR (n = 35,559), AFR (n = 1,871), EAS (n = 1,823). Sample size of the T2D GWAS meta-analysis used as outcome datasets: EUR (n = 242,283 cases and n = 1,569734 controls), AFR (n = 50,251 cases and n = 103,909 controls), AMR (n = 29,375 cases and n = 59,368 controls), EAS (n = 88,109 cases and n = 339,395 controls). Abbreviations: QTL=Quantitative trait loci; OR=Odds ratio; SD=Standard deviation; CI=Confidence Interval; PPH4=Posterior probability of hypothesis four. Source data

To detect potential causal effects that could be shared across genetic ancestry groups, we performed a meta-analysis of the single-ancestry MR causal estimates. Identifying shared effects that could potentially be targeted by drugs would increase the equitability of benefits across populations, a major current challenge for the translation of genomics findings into the clinic^40^. A total of 13,878 genes and 1,094 proteins had IVs in at least two genetic ancestry groups and were meta-analysed using random effect models to obtain a cross-ancestry causal effect estimate. We identify causal effects of 81 genes and five unique proteins on T2D risk in the MR meta-analysis across genetic ancestry groups (Extended Data Fig. 4 and Supplementary Tables 2 and 3). Most of these causal effects of genes (93.8%) and proteins (80%) were also detected in the EUR single-ancestry MR analysis, highlighting the driving power of this genetic ancestry group in the meta-analysis, linked to the larger sample size of the corresponding QTL and GWAS datasets (Fig. 4a). The meta-analysis identified four genes not detected in any single-ancestry MR analyses: *MUTYH*, *NEIL1*, *ZFP36L2* and *TUFM* (Fig. 4b). For *MUTYH* and *NEIL1*, the meta-analysis was driven by the statistical power in EUR (Fig. 4b). For *ZFP36L2* and *TUFM*, all genetic ancestries presented limited statistical power and similar estimates of causal effects, highlighting the power gain of meta-analysing MR results from multiple genetic ancestry groups.

Of the 81 genes with significant causal effects on T2D risk in the meta-analysis, evidence of ancestry-related heterogeneity, as determined by Cochran’s *Q* statistic, was found for none of the proteins and for only one gene, *KLHL42*, albeit with a *P* value very close to the nominal significance threshold (Cochran’s *P* = 3.96 × 10^−2^). Together with the single-ancestry analyses, these results indicate low levels of ancestry-related heterogeneity of causal effects of gene and protein expression levels on T2D risk.

### Causal effects present high tissue-related heterogeneity

**Fig. 5:**
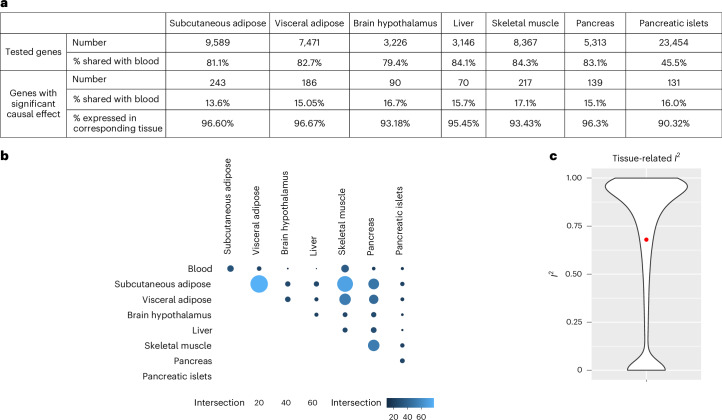
Overview of the results from the eQTL EUR MR analyses in T2D-relevant tissues. **a**, Number of genes tested in MR analyses from T2D-relevant tissues with significant causal effects on T2D risk, percentage of causal effects also detected in blood eQTL MR and percentage of causal effects detected in a tissue where the gene is also expressed. **b**, Pairwise overlap of significant causal effects across T2D-relevant tissues and blood eQTL MR. For easier reading, only pairwise overlap between different tissues is represented. **c**, Distribution of *I*^2^ values representing the heterogeneity of causal estimates for genes tested in at least two tissues (including blood). Source data

**Extended Data Fig. 5:**
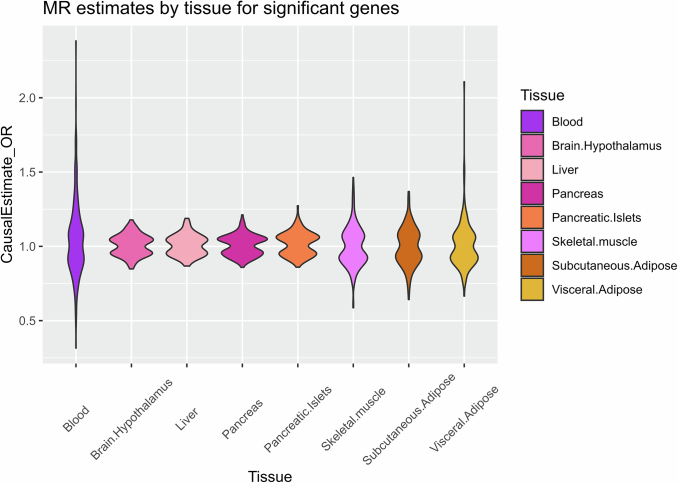
Distribution of causal effect estimates from the eQTL EUR MR analyses according to tissue. Only estimates of genes with causal effects detected in our analysis have been included. Source data

To increase the granularity of our causal inference analyses and the likelihood of detecting causal effects relevant for T2D^15^, MR analyses were conducted in EUR in seven tissues relevant to T2D: subcutaneous adipose, visceral adipose, brain hypothalamus, liver, skeletal muscle, whole pancreas and pancreatic islets. Causal effects on T2D risk were identified for 70 to 243 genes, depending on the tissue (Fig. 5a and Supplementary Table 7). More than 90% of the causal effects were identified in tissues in which the corresponding gene is also expressed, highlighting the validity of our agnostic gene-centric approach (Fig. 5a and Methods). One example is *MTNR1B*, a gene found to be impaired in early insulin secretion and expressed mostly in pancreatic islets^41^, the only tissue tested in our study with a strong causal effect (*q* = 2.17 × 10^−97^, PPH4 = 1; Supplementary Fig. 1). Given that molecular QTL data in blood are more widely available across multiple genetic ancestry groups and with higher sample sizes than in other tissues, we compared findings from non-blood tissues to the 335 genes identified in any blood MR analyses. A total of 928 genes showed significant causal effects on T2D risk in at least one T2D-relevant tissue and/or in blood. We observed the largest causal effects in blood, followed by visceral adipose tissue, skeletal muscle and subcutaneous adipose tissue (Extended Data Fig. 5).

We report 328 genes (36%) with significant causal effects in at least two tissues (Fig. 5b). Only 18% of the genes with causal effects identified in a T2D primary tissue also showed significant causal effects in blood, highlighting the additional information provided by eQTL MR analyses in non-blood tissues. The highest overlap was observed between subcutaneous and visceral adipose tissues, as well as between subcutaneous adipose tissue and skeletal muscle. We observed low overlap between whole pancreas and pancreatic islets, underscoring the importance of investigating fine-scale tissue molecular profiles. Only 28 out of the 328 genes with causal effects on T2D risk in multiple tissues showed concordant directions of effect across tissues. Accordingly, we observed a large tissue-related heterogeneity, as highlighted by the high *I*^2^ values depicted in Fig. 5c, which represents the percentage of variance caused by study heterogeneity. Among the genes tested in at least two tissues, 75% (14,304 out of 19,020) show nominally significant heterogeneity. This tissue-related heterogeneity could be underestimated given the lack of shared IVs across tissues when investigating *cis-*eQTLs^13^, with 11,707 genes (21%) in our analyses being tested in only one non-blood tissue. Evaluating the causal effects of all genes in disease-relevant tissues will be needed to comprehensively capture tissue-related heterogeneity and characterize shared and tissue-specific causal mechanisms.

### Contextualizing causal effects into the T2D landscape

**Fig. 6:**
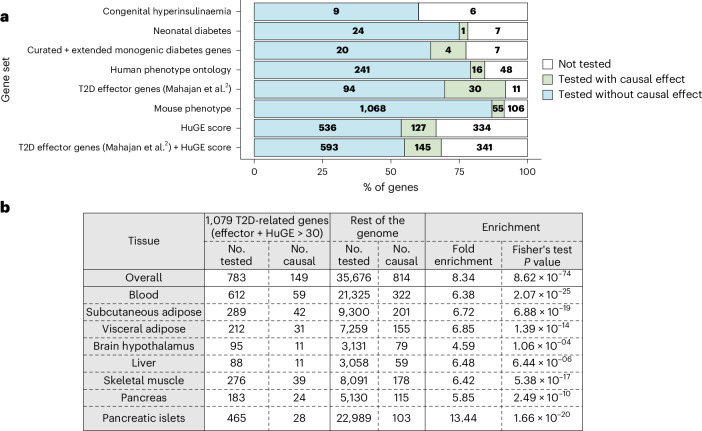
Causal effects among established diabetes-related gene sets. **a**, Stacked bars showing the number of genes not tested, or tested with and without causal effects across molecular traits. A gene was considered to have causal effects if it had a *q* value of <0.05, passed sensitivity analyses and showed evidence of colocalization (PPH4 > 0.8). Bars are ordered by the number of genes with causal effects. **b**, Enrichment of established 1,079 T2D effector genes (Mahajan & McCarthy^42^) and genes with HuGE scores of >30. The table shows the number of genes tested; that is, those with at least one IV, as well as the number of genes with causal effects for the list of 1,079 T2D effector genes and for the rest of the genome. The table also shows the fold enrichment of the percentage of causal effects among tested genes and the corresponding *P* value from Fisher’s exact test (two-sided). Results are reported overall and by tissue. Source data

In this study, we performed a gene-centric agnostic approach; that is, without being constrained to T2D loci. To validate this approach in light of previous T2D findings, we compared the molecular traits with causal effects on T2D risk from this study to established diabetes-related effector genes, using eight different gene sets (Methods and Supplementary Table 9). Between 8% and 40% of the diabetes-related genes could not be tested in any of our MR analyses, depending on the gene set considered (Fig. 6a). The proportion of genes with a causal effect on T2D risk identified among those tested increased with the level of supporting evidence for T2D, ranging from less than 5% in congenital and neonatal diabetes forms to 20% or more when focusing on T2D effector genes as defined by Mahajan & McCarthy^42^ and Human Genetic Evidence Calculator (HuGE) scores^38^ (Fig. 6a and Supplementary Table 10).

**Extended Data Fig. 6:**
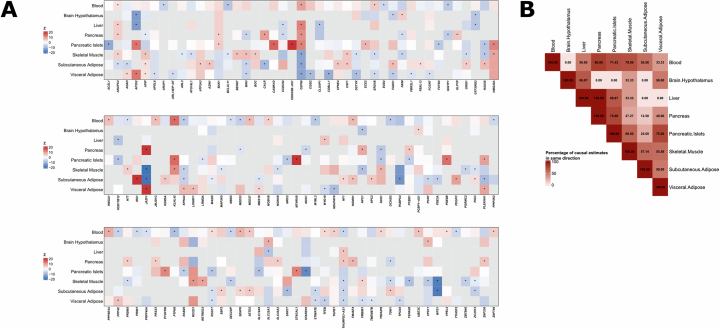
Causal effects of diabetes-related genes in the different tissues. (**A**) Z-scores of the causal estimates of the DM-related genes with causal effects in at least one of the MR analyses. Grey boxes correspond to genes not tested in the corresponding tissue, and boxes with a star indicate genes for which we define a causal effect on T2D risk. For blood, the z-score corresponds to the most significant analysis from eQTL and pQTL MR, regardless of the ancestry. **(B)** Heatmap representing the percentage of causal effects with a concordant direction between pairwise tissues. Only the pairs of genes/tissues with causal effects have been included. Source data

To extend these analyses, we included the list of 1,079 T2D genes using the combined definition of T2D effector genes from Mahajan & McCarthy^42^ and HuGE scores of >30, representing the gene set with the highest number of curated T2D-relevant genes with causal effects^38^. For this gene set, we showed an enrichment of causal effects compared to the rest of the genes tested in our analyses: causal effects were identified for 19.03% of T2D-related genes, but for only 2.28% of the rest of the genes (*P* = 8.62 × 10^−74^; Fig. 6b and Methods). The same trends were found when considering causal effects at the single-tissue level (*P* values ranging from 1.06 × 10^−4^ to 2.07 × 10^−25^). When investigating the pattern of association of tested T2D-related genes in the different tissues, we observed that strong effects were often found in pancreatic islets (Extended Data Fig. 6a), most of the time in a similar direction as in pancreas (Extended Data Fig. 6b). On the contrary, the direction of causal effect in pancreatic islets was opposite to the one observed in subcutaneous tissue for 75% of the genes with causal effects across the two tissues. For example, this is the case for *BAK1* (coding a pro-apoptotic protein from the BCL-2 family), which is significant and passes sensitivity analyses in all tissues, but is confirmed by colocalization evidence only in pancreas and pancreatic islets. Based on MR causal estimates, increased expression of this gene is associated with decreased T2D risk in skeletal muscle and adipose tissues, with increased risk in all the other tissues tested.

**Extended Data Fig. 7:**
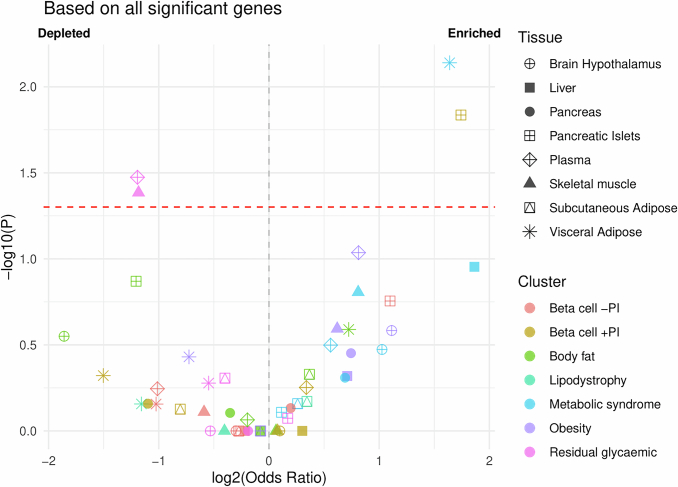
Volcano plot of the OR contrasting cluster membership with evidence of causal effect. The p-value for the Fisher’s exact test (two-sided) is reported, with the horizontal dashed line corresponding to the nominal significance threshold. Source data

To further investigate how tissue-informed causal inference analyses can shed light on established T2D genes, we compared our findings to the eight mechanistic clusters derived in previous work^1^ (Methods, Extended Data Fig. 7 and Supplementary Table 11). Genes assigned to the metabolic syndrome clusters were more likely to be causal in visceral adipose tissue, with visceral adiposity being a main driver of the metabolic syndrome^43^ (Fisher’s *P* value = 7.23 × 10^−3^, OR = 3.12). Similarly, genes assigned to the beta cell PI+ cluster were more likely to show evidence of a causal effect in pancreatic islets (Fisher’s *P* value = 1.46 × 10^−2^, OR = 3.35). These examples show that pinpointing the tissues driving causal effects can help unravel the molecular mechanisms of T2D heterogeneity.

### eQTL and pQTL analyses offer complementary and non-redundant insights

To assess the complementarity between eQTL and pQTL MR findings, we compared the genes and proteins with significant causal effects identified by each analysis (Table 1).

**Table 1:** Molecular traits (genes and proteins) with evidence of causal effects on T2D risk from eQTL and pQTL MR

		eQTL							
		Blood	Subcutaneous adipose	Visceral adipose	Brain hypothalamus	Liver	Skeletal muscle	Pancreas	Pancreatic islets
	No. of significant causal effects	335	243	186	90	70	217	139	131
**pQTL**	EUR (*n* = 39)	HIBCH*	CPXM1* HIBCH* HYAL1	HIBCH*	-	GSTA1*	CPXM1* HIBCH*	GSTA1*	PTGFRN HIBCH*
	AFR (*n* = 0)	-	-	-	-	-	-	-	-
	EAS (*n* = 8)	-	-	-	-	-	BOC FAM20B	-	-
	Meta-analysis (*n* = 5)	-	-	-	-	-	-	-	-

^*^Replicated in at least one independent ancestry-matched cohort; that is, with *q* < 0.05 in the replication cohort and concordant direction of effect with the discovery cohort

**Fig. 7:**
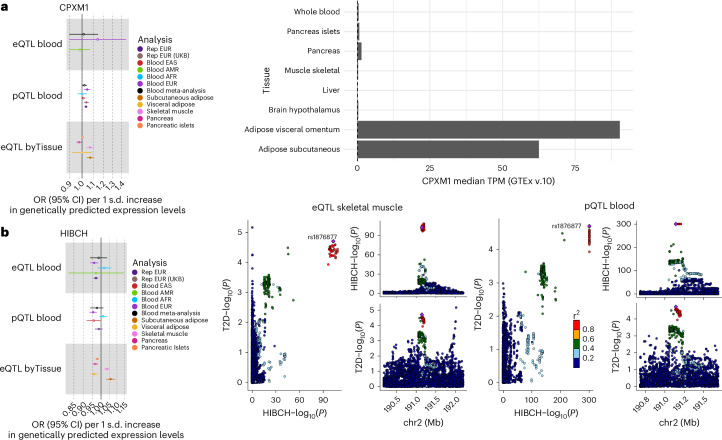
Results for CPXM1 and HIBCH. **a**,**b**, For each molecular trait, causal estimates from blood eQTL, plasma pQTL and T2D-relevant tissue eQTL MR analyses are shown for CPXM1 (**a**) and HIBCH (**b**). Filled dots represent causal estimates from MR analyses that have a *q* value <0.05 and (1) pass the sensitivity criteria and show evidence of colocalization (PPH4 > 0.8) in single-ancestry analyses or (2) present nominal significance and meet criteria (1) in at least one cohort entering the meta-analysis. Points represent MR causal estimates derived from summary statistics (OR for T2D per s.d. change in genetically predicted gene expression or protein levels, a measure of centre); error bars, 95% CI. Sample sizes of the QTL datasets used as exposure datasets: (1) eQTL EUR (discovery *n* = 31,684, replication *n* = 801), AFR (discovery *n* = 1,032, replication *n* = 757), AMR (discovery *n* = 893, replication *n* = 784); (2) pQTL EUR (discovery *n* = 35,559, replication ARIC *n* = 7,123, replication UKB *n* = 54,219), AFR (discovery *n* = 1,871, replication AASK *n* = 466, replication UKB *n* = 262), EAS (discovery *n* = 1,823, replication UKB *n* = 262). Sample size of the T2D-relevant tissues eQTL: subcutaneous adipose (*n* = 711), visceral adipose (*n* = 584), brain hypothalamus (*n* = 256), liver (*n* = 261), skeletal muscle (*n* = 816), pancreas (*n* = 362), pancreatic islets (*n* = 446). Sample size of the T2D GWAS meta-analysis used as outcome datasets: EUR (*n* = 242,283 cases and *n* = 1,569,734 controls), AFR (*n* = 50,251 cases and *n* = 103,909 controls), AMR (*n* = 29,375 cases and *n* = 59,368 controls), EAS (*n* = 88,109 cases and *n* = 339,395 controls). The median TPM observed in GTEx in the eight tissues tested in our MR analysis is represented for CPXM1. For HIBCH, the LocusCompare and LocusZoom plots demonstrating the colocalization evidence from eQTL in T2D-relevant tissues are displayed. ARIC, Atherosclerosis Risk in Communities study; UKB, UK Biobank; AASK, African American Study of Kidney Disease and Hypertension; TPM, transcripts per million. Source data

The absolute number of molecular traits with overlapping evidence of causal effects of gene expression and protein abundance levels was small (seven out of 1,563 molecular traits tested in both eQTL and pQTL MR analyses), in line with the expected complementary information captured by the two molecular levels^44^. One example is CPXM1, for which we identified causal effects on T2D risk in the plasma pQTL MR analysis (OR = 1.05, *q* = 1.45 × 10^−2^), replicated in two independent cohorts from EUR, as well as in the eQTL MR analysis in skeletal muscle (OR = 1.07, *q* = 1.23 × 10^−4^) and subcutaneous adipose tissue (OR = 1.07, *q* = 5.69 × 10^−5^), always with increasing expression levels associated with increased T2D risk (Fig. 7a). *CPXM1* has a role in adipose tissue production^45^ and is associated with insulin resistance in polycystic ovary syndrome^46^. *CPXM1* is expressed in multiple tissues, especially in subcutaneous adipose tissue, but tissue-specific pQTL data will be needed to assess the origin of circulating CPXM1 abundance. Only *HIBCH* was identified in eQTL-based and pQTL-based causal inference analyses in blood (eQTL OR = 0.96, *q* = 1.37 × 10^−2^; pQTL OR = 0.95, *q* = 4.94 × 10^−4^). Increased expression levels of this gene in pancreatic islets (OR = 0.98, *q* = 2.9 × 10^−3^) and visceral adipose tissue (OR = 0.96, *q* = 1.15 × 10^−3^) also showed a significant protective causal effect against T2D risk (Fig. 7b). By contrast, higher expression levels of *HIBCH* in subcutaneous adipose tissue (OR = 1.06, *q* = 5.68 × 10^−4^) and skeletal muscle (OR = 1.04, *q* = 5.31 × 10^−4^) were causally associated with increased T2D risk.

Six additional molecular traits presented significant causal effects on T2D risk in both eQTL from T2D-relevant tissues and in the blood pQTL MR analyses. Although we investigated non-blood tissue MR in EUR only, owing to data availability, we found overlap with blood pQTL findings from EAS for *FAM20B* and *BOC*. Future studies are needed to characterize non-blood molecular QTLs in non-EUR to validate these results. Obtaining non-blood QTLs in global populations will also provide insights into the ancestry-related heterogeneity in non-blood tissues.

For subcutaneous adipose tissue, skeletal muscle and liver, we found a significant enrichment of overlapping molecular traits with causal effects in blood eQTL and pQTL causal inference analyses (Methods). This highlights the non-redundant information captured by gene expression and protein abundance levels, for example, owing to post-transcriptional changes not captured at the RNA level^44^ (Methods and Supplementary Table 8).

## Discussion

In this study, we unravel causal molecular mechanisms influencing T2D risk in an ancestry-aware and tissue-aware manner. To our knowledge, this represents the most comprehensive multi-ancestry analysis of the causal effects of genes and proteins on T2D risk, including the use of EAS pQTL data, which was not included in earlier studies. In total, we have identified 963 molecular traits with a causal effect on T2D risk in at least one tissue. The causal effects of 79 gene and protein expression levels in blood were replicated in independent cohorts from a similar genetic ancestry group, including previously described T2D-related genes, and unreported potential effector genes such as *CPXM1* and *HIBCH*. With only a few exceptions, such as *PTGES2* and *TOLLIP-AS1*, we identified most causal effects in EUR and observed low ancestry-related heterogeneity, suggesting shared effects across genetic ancestry groups. However, this observation needs to be confirmed in future studies once equivalent statistical power is achieved across genetic ancestry groups (Extended Data Fig. 1). Considerable effort will be required to meet this goal, as most molecular QTL studies are typically under-represented in non-EUR genetic ancestry groups^22^. Conversely, we observed high heterogeneity of causal effects across tissues, including opposite directions of effect. This was observed even between anatomically similar tissues, such as pancreatic islets and the whole pancreas, potentially suggesting cell-type-specific causal effects that cannot be detected from tissue-level QTL data. Additionally, 85% of the causal effects of gene expression detected in T2D-relevant tissues were not detected in blood. This highlights the need to continue increasing the granularity in measuring gene expression levels across tissues and at the single-cell level to understand biological processes leading to the development of diseases.

Non-EUR QTL data availability remains a limitation of the present study. Causal inference analyses were restricted to three non-EUR genetic ancestry groups (AMR in eQTL, EAS in pQTL MR and AFR in both omics levels), which were limited in statistical power for most of the molecular traits compared to EUR. It is therefore possible that non-EUR MR analyses include false negatives. However, this is less problematic than drawing false positive conclusions of causality and should improve as QTL studies become larger and more diverse in the future^22^. We show through the example of *TUFM* that performing meta-analysis across single-ancestry MR estimates enables us to identify causal effects of traits with limited statistical power but concordant effects across ancestries. *TUFM* has a suggested role downstream of the insulin cascade^47^ and its genetic expression might be impacted by a genetic inversion associated with obesity^48^. The lack of diversity in QTL maps is even stronger at the scale of T2D-relevant tissues, where the present analyses were restricted to EUR at the eQTL level. Sourcing pQTL data from primary tissues would be of interest to better understand causal mechanisms at the protein level and to assess whether we recapitulate the causal effects identified at the gene expression level. It is, however, likely that causal effects would be different between the two omics levels, given their distinct genetic regulation^49^ and the moderate correlation between gene expression and protein levels, impacted by post-transcriptional steps^50^. This is consistent with the low overlap of traits with causal effects identified in our analysis between the eQTL and pQTL MR. Data limitation also lies in the design of omics assays themselves, which are currently restricted to a set of given proteins with predicted impacts on human health. Proteomics platforms such as Olink and Somascan vary in their selection of proteins and quantification technologies, potentially leading to low correlations for the same protein and the subsequent description of their genetic regulation, especially *trans* effects^51,52^. This limits the possibility of replication between independent studies assayed on different platforms.

**Extended Data Fig. 8:**
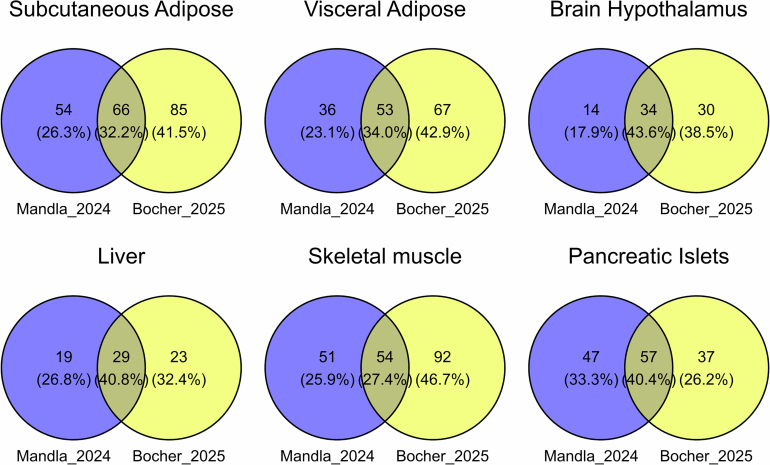
Comparison of causal genes with previous colocalization effort. Venn Diagrams representing the overlap of genes identified in the previous colocalization study from Mandla et al. 2024, and in the present study (MR+colocalization using PWCoCo) are indicated for six tissues which used the same QTL datasets in both studies. Source data

The molecular causes modifying T2D risk in the current study are solely identified based on computational evidence, with the main challenge being to establish whether they reflect true biological effects. To minimize the risk of identifying false positives, we highlight key methodological aspects to be integrated into similar studies for complex traits. This includes the importance of accounting for complex polygenic architectures of diseases using dedicated methods such as the colocalization approach PWCoCo^53^ and using ancestry-matched summary statistics. For instance, when comparing the genes tested in our study with those tested in the most recent large-scale colocalization work based on the coloc method using the same QTL datasets^23^, we describe more colocalization signals for all investigated T2D-relevant tissues (Extended Data Fig. 8). The additional signals detected in our study map to loci with complex linkage disequilibrium patterns, such as *C1GALT1* (Supplementary Fig. 2). Similarly, a prior pQTL MR study on T2D risk described causal effects for 11 proteins using the same pQTL deCODE dataset and similar criteria^9^. Our analytical approach replicated seven of these causal effects and identified additional causal effects for 39 proteins. Validating MR results through sensitivity analyses and colocalization is needed to avoid declaring false positive results. In our study, increasing the number of criteria to declare significant MR results led to an increased replication rate (Supplementary Table 12), in line with previous recommendations^4^. Despite using a strict strategy to define putative causal effects, the replication rate among genes that could be tested in independent cohorts remained lower than 50%, potentially because of violations of MR assumptions or power. Additionally, we note that only a few traits could be verified for sensitivity analyses, as most of the genes and proteins were instrumented by only one *cis-*QTL. We used an IV selection strategy based on the *F*-statistics and a *cis*-window definition (which varies across studies) to limit departures from the relevance and exclusion restriction assumptions, respectively. However, the robustness of our results to the *cis*-windows definition should be validated in future studies, as larger windows are more likely to capture pleiotropic variants regulating other genes. We therefore advocate for careful interpretation of MR results and the use of replication approaches, which will be needed to validate the potential candidates from our non-blood MR results. However, replication is currently challenged by the lack of common instrumented molecular traits between the discovery and replication cohorts.

We show an enrichment of causal effects identified among genes expected to be associated with T2D, confirming that the above methodological steps enable us to identify molecular traits relevant to T2D using our gene-centric approach. However, a substantial fraction of T2D-related genes did not show causal effects in our analyses, as a result of several factors. Firstly, very stringent criteria have been used to define causal effects. For example, the proportion of causal effects among tested genes jumps from 19.6% to 49.7% for curated T2D-related genes when considering only MR significance. This is the case for *FTO*, for example, which is highly significant in skeletal muscle eQTL MR (*q* = 4.62 × 10^−106^), but not confirmed by colocalization (PPH4 = 0.16). Secondly, many T2D-related genes could only be tested in a few tissues because of IV availability. Given the high tissue-related heterogeneity identified in our study, it is possible that even if a gene had been tested, it was not in the relevant tissue or at the relevant omics level. Thirdly, there can be ambiguity around the gene causal for T2D in a given locus. One-third of the loci in which a causal effect was identified overlap with one of the 1,079 predicted T2D effector genes, highlighting the difficulty in disentangling causal mechanisms in a genomic region (Supplementary Fig. 3). Furthermore, the list of 1,079 curated T2D genes corresponds to genes which have been associated with T2D, but these associations may reflect correlation or downstream consequences rather than a direct causal effect on disease risk, underscoring the value of our MR-based comparison to prioritize likely causal genes. Finally, several studies have shown that variants identified in GWAS and eQTL studies present different characteristics, such as in their regulatory landscape or selective pressure^54^. QTLs from primary tissues might not be the optimal IVs to model the causal effect of molecular traits on disease risk, which will hopefully be improved in the future, in particular by increasing their granularity in single-cell investigation.

Even when limiting the risk of declaring false positives, an additional key challenge of computational-based causal inferences lies in the biological interpretation of identified causal effects. Despite this limitation, we were able to show that the tissue in which causal effects of T2D effector genes were identified aligns with T2D biology heterogeneity captured by the clusters defined in the latest T2D GWAS meta-analysis^1^. In this work, we provide additional insights into genes already predicted to be associated with T2D, such as *BAK1*, a pro-apoptotic gene. Its pro-apoptotic role has been described in pancreatic β cells in mice^55^, with apoptosis being one of the causes of loss of β cells in individuals with T2D, in line with the observation in our study that increased expression of this gene in the pancreas and pancreatic islets increases T2D risk. Conversely, increased levels of *BCL-2* have been associated with decreased body mass index in obese patients^56^*.* Given that *BAK1* is a member of the BCL-2 family, these previous studies are consistent with a protective role of increased *BAK1* expression on T2D risk identified in adipose tissues in our study. This example highlights how increased expression levels in different tissues can have opposite effects on T2D risk. We also describe a causal effect of ALDH2 abundance on T2D risk in EAS, for which the IV rs4646776 has been associated with drinking behaviour in this population^57^, with alcohol being a causal risk factor for T2D^58^. Previous studies have shown that rs4646776 is in high linkage disequilibrium with rs671, which is associated with decreasing ALDH2 stability and activity, further leading to reduced alcohol consumption^59^. This is an example of vertical pleiotropy, whereby lower alcohol consumption is thought to be a consequence of reduced functional ALDH2, not invalidating MR assumptions and our conclusions on the causal effects of ALDH2.

In addition to identifying previously curated T2D effector genes, we suggest additional genes to prioritize for future studies. One example is *HIBCH*, for which we observed causal effects in different directions depending on the tissue, with effects in the blood replicated in an independent cohort. These results complement previous findings in which causal effects of this gene have been reported in EUR from blood eQTL and pQTL analyses^60^, as well as in the liver, pancreas and visceral adipose tissue^61^, all with protective effects against T2D, a direction concordant with our study. However, the biological interpretation of these opposite effects across tissues is much more challenging to understand in the context of T2D and will require more comprehensive data and additional investigation. It is important to note that although challenging to interpret, opposite directions of effects across tissues are expected and have been observed in other studies, such as the effect of *SORT1* on low-density lipoprotein levels^16^.

In summary, we have conducted, to our knowledge, the largest multi-tissue and multi-ancestry multi-omics causal inference analysis of T2D to date. We identify 923 genes and 46 proteins for which expression levels show a causal effect on T2D risk. These findings expand the catalogue of putative causal molecular traits and effector genes influencing T2D. By providing replication in independent cohorts and comparison of findings across ancestries, tissues and molecular levels, we provide strong causal candidates modulating T2D risk that may generalize to many diverse global populations. Our findings help prioritize genes and proteins for functional validation in future experimental studies and investigation as molecular targets for T2D treatment or prevention.

## Methods

### Study design overview

In this study, we performed blood MR analyses by using IVs defined from blood eQTLs and plasma pQTLs from multiple cohorts of four large populations in an ancestry-aware manner. Causal effects were considered based on MR statistical significance corrected for multiple testing, sensitivity analysis and colocalization, as described in more detail below. Replication was performed in matched genetic ancestry groups, depending on data availability. We meta-analysed MR results across genetic ancestry groups for either genes or proteins, separately. We also performed *cis-*eQTL MR analysis in seven additional T2D-relevant tissues. All datasets are reported in Supplementary Table 1 and explained in more detail in the following sections.

### Datasets

All contributing cohorts have ethical approval from their institutional ethics review boards. We performed two-sample MR using eQTL as well as pQTL datasets of various genetic ancestry groups and tissues (Fig. 1 and Supplementary Table 1). Blood eQTL data were available in EUR, AFR and AMR, while plasma pQTL data were available for EUR, AFR and EAS. Although genetic ancestry is on a continuous scale, we defined genetic ancestry groups based on 1000 Genomes Project phase 3 individuals as a reference ^18^. GWAS summary statistics for T2D, the outcome in our MR analyses, correspond to the T2DGGI GWAS summary statistics^1^ from the same genetic ancestry group as the QTL datasets used as the exposure. When multiple QTL datasets were available for the same genetic ancestry group, we chose to maximize the sample size of the discovery cohorts. This led to the inclusion of eQTLGen^26^ for discovery and GENOA^28^ for replication in the eQTL EUR MR, and deCODE^27^ for discovery and ARIC^29^ for replication in the pQTL EUR MR. Additionally, we performed eQTL MR on seven further T2D-relevant tissues: subcutaneous adipose tissue, visceral adipose tissue, liver, brain hypothalamus, skeletal muscle, pancreas (all coming from GTEx^34^) and pancreatic islets (TIGER^35^). Owing to data availability, these analyses were restricted to EUR, with T2DGGI GWAS summary statistics specific to EUR being used for the outcome data.

### MR

#### MR assumptions

MR relies on three core assumptions, namely the relevance, independence and exclusion restriction assumptions. The relevance criteria assume that the IVs selected to run the MR analyses should be strongly associated with the exposure, that is, being strong predictors of the exposure. This assumption can be directly tested in MR, as the strength of association between genetic variants and exposure can be computed using the *F*-statistic, defined as $F=\frac{{\beta }^{2}}{{\mathrm{se}}^{2}}$, where *β* corresponds to the effect size estimate of the association between the genetic variant and the gene expression or protein abundance level, and se corresponds to its standard error. The independence assumption states that there are no confounders of the IV–outcome relationship. The exclusion restriction assumption ensures that there is no horizontal pleiotropy; that is, that the effect of the IVs on the outcome is mediated solely through the exposure. Neither assumption can be directly tested in MR analyses, but sensitivity methods (detailed below) that relax MR assumptions can be used to limit potential biases.

### Selection of IVs

We selected IVs for gene expression and protein abundances from *cis*-QTLs by using the *cis*-windows and the significance threshold defined in each study (Supplementary Table 1). To select the IVs to be used in MR, we first performed a linkage disequilibrium clumping using PLINK (v.1.9)^62^ and the default parameters recommended in the TwoSampleMR package^63^; that is, in a 10 Mb region considering an *r*² threshold of 0.001, with the 1000 Genomes Project phase 3 (ref. ^18^) from the corresponding genetic ancestry group being used as the reference panel. If any IV was not present in the T2DGGI GWAS summary statistics, we replaced it with a linkage disequilibrium-based proxy (*r*² > 0.8) using the ieugwasr::ld_matrix() R function (v.1.0.2) and the output from PLINK (v.1.9)^62^, which clumps variants into clusters. We then selected variants with an *F*-statistic greater than ten to limit weak instrument bias^64^ and verify the MR relevance assumption.

### Single-ancestry two-sample MR analyses

All MR analyses were performed using the TwoSampleMR package^63^ (v.0.5.9). Alleles were harmonized between the exposure and outcome data using the function harmonise_data() with default parameters. We report causal estimates as OR per unit of genetically predicted gene expression and protein levels on T2D risk. Several criteria were used to define causal effects as previously recommended for *cis*-MR analyses^65^: (1) false discovery rate (FDR)-adjusted *P* values (*q* values) lower than 5% from the inverse variance weighted (IVW) method, or from the Wald ratio if only one IV was present (FDR correction was applied within each cohort). (2) Concordant effect across four sensitivity analyses: weighted median, MR-PRESSO^66^ and MR-Egger^67^ to test for pleiotropy, as well as Steiger-filtered IVW. If the distortion test of MR-PRESSO was significant (*P* < 0.05), we considered the effect estimate of the outlier-corrected method. Otherwise, we used the estimate of the raw MR-PRESSO method. Steiger-filtered IVW was used to limit for reverse causation; that is, when the direction of the causal association is actually from the outcome to the exposure. In our study, T2D represents the exposure when using the Steiger filtering, which relies on the liability model and assumption of T2D prevalence, which we set at 10% (ref. ^68^). (3) Showing no significant heterogeneity (heterogeneity *I*^2^ lower than 50%) and pleiotropy (MR-Egger intercept test *P* < 0.05). (4) To limit the risk of false positives that could arise as a result of violations of the exclusion assumption in MR^65^, and especially bias owing to potential horizontal pleiotropy, we performed colocalization using PWCoCo^53^, only retaining genes and proteins with a posterior probability of a shared causal variant (that is, PPH4) greater than 0.8. We note that PWCoCo first performs standard colocalization using coloc, and only when PPH4 < 0.8 does it proceed to colocalization on secondary genetic associations, found through conditional analyses. The validation of MR results by colocalization is also important to reduce the risk of bias caused by violation of the independence assumption.

Although overlap of exposure and outcome data is possible in our two-sample MR analyses, we expect the impact to be limited owing to the selection of IVs highly predictive of the exposure and the high sample sizes^69^.

### Replication in independent cohorts

We tested the genes and proteins with significant causal effects in the discovery analyses for replication in an independent cohort from the same genetic ancestry group (Fig. 1 and Supplementary Table 1). For the pQTL analyses, no replication cohort with the same SomaLogic platform was available in EAS. In addition to using SomaLogic replication cohorts for EUR and AFR, we used the UK Biobank cohort for all populations (EUR, AFR and EAS)^36^, for which proteins were assayed using the Olink panel. We declared causal effects of genes and proteins as replicated if they showed a *q* value lower than 5% (the correction being applied among the genes and proteins with significant causal effects identified in the discovery analyses and that could be tested in the replication set) and a concordant direction of effect with the IVW estimate from the discovery cohort.

### Multi-ancestry MR meta-analysis

In addition to performing single-ancestry MR, we conducted eQTL and pQTL multi-ancestry MR meta-analyses for molecular traits that could be tested in at least two ancestries using the metafor package^70^ (v.4.6). We used IVW with a random-effect model. We defined genes and proteins with significant causal effects if they presented a *q* value lower than 5% in the meta-analysis and had compelling evidence in at least one cohort; that is, with a nominally significant *P* value, along with the MR sensitivity and colocalization criteria met in at least one of the entering cohorts.

### Follow-up analyses

#### Replication rate according to MR significance criteria

We evaluated the impact of a stricter definition of significant causal effects in our MR analyses on the replication rate by using the blood eQTL EUR data as an example, as it presents the largest number of significant findings (eQTLGen as the discovery cohort and GENOA as the replication cohort). We computed the replication rate by defining significant signals in the discovery cohort using five criteria: (1) all tested genes as significant; (2) genes with a nominally significant *P* value; (3) genes with a *q* value lower than 5%; (4) genes from criterion 3 with a concordant direction of effect with sensitivity analyses, no heterogeneity and no pleiotropy; and (5) genes from criterion 4 with evidence from colocalization (PPH4 > 0.8). For the latter, we recomputed the replication rate by distinguishing genes with only one IV from genes with more than one IV, given that no sensitivity methods can be applied if only one IV is present. Replication was tested for the significant causal effects in the discovery cohort, and the replication rate was computed as the proportion of genes with a *q* value lower than 5% in the replication cohort among the tested genes for each replication criterion. The FDR correction in the replication cohort was applied to the set of significant genes from the discovery cohort that could be tested in the replication cohort.

### Investigation of tissue gene expression

To assess the tissues in which genes were expressed, we used RNA sequencing gene expression data from GTEx (v.10) (https://storage.googleapis.com/adult-gtex/bulk-gex/v10/rna-seq/GTEx_Analysis_v10_RNASeQCv2.4.2_gene_tpm.gct.gz). We considered a gene to be expressed in a tissue if the median value of the transcripts per million was higher than 0.1. We focused on the tissues included in this study (blood, subcutaneous adipose tissue, visceral adipose tissue, brain hypothalamus, liver, skeletal muscle, pancreas, pancreatic islets).

### Computation of minimal detectable effect

We approximated the statistical power of MR analyses by computing the minimal detectable effect (MDE) for each gene that can be detected with a power of 80%. It is based on the same calculations as in https://shiny.cnsgenomics.com/mRnd, detailed in a previous publication^71^. In brief, MR statistical power depends on the proportion of variance in the exposure explained by the IVs (*R*^2^), the causal effects of the exposure on the outcome (*b*_MR_), the sample size of the outcome (*n*) and the proportion of cases (*K*) for binary traits. To derive the statistical power of a given MR analysis, the non-centrality parameter (NCP) is computed based on the formula:

$$\mathrm{NCP}=\frac{{b}_{\mathrm{MR}}^{2}}{\mathrm{var}({b}_{\mathrm{MR}})}$$

*b*_MR_ and var(*b*_MR_) are not known, and are estimated as:

$${b}_{\mathrm{MR}}=K\left(\frac{\mathrm{OR}}{1+K\left(\mathrm{OR}-1\right)}-1\right)$$

with OR being the true odds ratio of the exposure on the outcome.

$$\mathrm{var}\left({b}_{\mathrm{MR}}\right)=\frac{K\left(1-K\right)\times {{b}_{\mathrm{MR}}}^{2}}{N\times {R}^{2}}$$

*R*^2^ can be estimated from the minor allele frequency of a given IV *i* (MAF_*i*_) and the estimate of the IV-exposure association (*b*_*E*,*i*_), summed over all the IVs of a given exposure as^72^:

$${R}^{2}=\sum 2\times {\mathrm{MAF}}_{i}\times \left(1-{\mathrm{MAF}}_{i}\right)\times {b}_{E,i}$$

Given that OR is not known, we cannot compute an expected power for a given MR analysis. We also cannot use the estimate from the MR analyses we performed, as the corresponding power would correspond to a post hoc power. Instead, we decided to compute the MDE to achieve a power of 80%. For this process, we computed the statistical power using the NCP and estimates from the different formulas using various theoretical values of OR and defined the MDE as the OR value for which a statistical power of at least 80% was achieved.

All analyses were run in R (v.4.3.3) using scripts that are available on GitHub: https://github.com/Ozvan/OmicsMR.

### Comparison of causal effects with T2D mechanistic clusters

To compare our findings to previous T2D knowledge, we used the eight mechanistic clusters defined in the latest T2D GWAS meta-analysis^1^. We determined the nearest gene for the 1,279 index variants from the GWAS meta-analyses using FUMA^73^. For each gene, we determined whether it was assigned to one of the eight mechanistic clusters from that GWAS meta-analysis^1^ (Supplementary Table 8 from the corresponding paper) and whether it was significant in our eQTL MR analyses from any tissue. We evaluated whether genes with causal effects identified in each tissue-specific MR analysis were enriched in any predefined clusters. For each tissue–cluster pair, we constructed a contingency table contrasting cluster membership with evidence of causal effect and retained only those with at least one overlapping gene. Enrichment was assessed using Fisher’s exact test. ORs and *P* values were extracted, and FDR correction was applied to account for multiple testing.

### Compilation of established diabetes-related genes

To investigate how our results map to the current T2D knowledge, we compiled eight sets of diabetes-mellitus-related genes based on multiple sources of evidence as described below (Supplementary Table 9):

1. Human Phenotype Ontology: genes defined by Human Phenotype Ontology terms, including insulin, diabetes, glucose and HbA1c. The Human Phenotype Ontology project provides a standardized ontology of medically relevant phenotypes and disease–phenotype annotations (https://hpo.jax.org).
2. Monogenic diabetes: genes harbouring mutations that cause monogenic diabetes from the Monogenic Diabetes Expert Panel (https://clinicalgenome.org/affiliation/50016/) and an extended list of syndromic monogenic genes^74^.
3. Congenital hyperinsulinaemia: genes harbouring mutations that cause congenital hyperinsulinism^75^.
4. Neonatal diabetes: genes harbouring mutations that cause neonatal diabetes (https://dnatesting.uchicago.edu/tests/neonatal-diabetes-panel).
5. Mouse phenotype: genes reported as relevant to T2D in mouse models, as previously reported^76^. Within the Mouse Genome Informatics Database (https://www.informatics.jax.org), we considered genes matching diabetes-relevant phenotypes under the broader category of ‘mouse phenotypes and mouse models of human disease’. For phenotypes associated with increased diabetes risk, we used impaired glucose tolerance, increased circulating glucose, insulin resistance and decreased insulin secretion. For phenotypes associated with decreased diabetes risk, we used improved glucose tolerance, decreased circulating glucose, increased insulin sensitivity and increased insulin secretion. For phenotypes related to diabetes risk but with unclear direction of effect, we used decreased circulating insulin and increased circulating insulin.
6. T2D predicted effector genes: T2D effector gene predictions (Mahajan & McCarthy^42^; https://t2d.hugeamp.org/research.html?pageid=mccarthy_t2d_247), which integrate genetic evidence, regulatory evidence from T2D or glycaemic trait-associated noncoding variants influencing gene expression in relevant tissues, and perturbation evidence (including phenotypes from gene perturbation in model organisms) to classify genes by their likelihood of being causal for T2D. We included genes under causal, strong and moderate evidence categories.
7. HuGE score ≥ 30: genes prioritized using HuGE^77^, a Bayesian framework that evaluates the extent to which human genetic data support the hypothesized involvement of a gene in disease mechanisms by integrating evidence from common and rare variation to generate a quantitative HuGE score. We applied the framework to T2D and included genes with a score of ≥30, corresponding to strong, extreme and compelling categories.
8. Combined list of T2D predicted effector genes and HuGE score ≥30: union of genes from criteria 6 and 7.

### Enrichment of causal effects

To assess whether there was a significant enrichment of causal effects detected in both eQTL and pQTL MR analyses, we performed a Fisher’s exact test. We compared the proportion of molecular traits tested in both eQTL and pQTL MR analyses to the proportion of molecular traits with significant causal effects in both eQTL and pQTL MR analyses. This test was performed for each T2D-relevant tissue and for blood.

A Fisher’s exact test was also used to assess the enrichment of causal effects within the list of established T2D effector genes, which were compared to the rest of the tested genes. We applied this test for molecular traits significant in any MR analysis, and by tissue.

### Investigating loci of genes with causal effects on T2D risk

We compared our set of identified T2D causal genes against the list of established T2D effector genes using the above criterion 8 (curated list by Mahajan & McCarthy^42^ and HuGE score of ≥30). We selected a 1 Mb window around each significant T2D causal gene and overlapped it with the curated lists of T2D effector genes. We divided the T2D causal genes into three groups: (1) genes that are in genetic loci without previously curated T2D effector genes; (2) genes that are included in the curated T2D effector genes list; and (3) genes that are in genetic loci where a different T2D effector gene was previously prioritized. To further investigate the genes in the latter group, we determined whether the curated T2D effector genes were tested in our MR analyses and expressed in the putative causal tissue.

### Comparisons with findings from the latest large-scale T2D colocalization study

We compared our findings to the results from the latest large-scale colocalization effort^23^, which used the T2DGGI GWAS multi-ancestry summary statistics^1^ and the coloc method^24^. The authors of that study used an approach to test for colocalization around the 1,289 T2D index variants. Our approach was centred on the molecular traits, as we investigated causal effects through MR, corroborated by colocalization for all the molecular traits that could be instrumented. We restricted our comparisons to the molecular traits tested in both studies. We only compared the analyses in T2D-relevant tissues across the two studies for subcutaneous adipose tissue, visceral adipose tissue, liver, brain hypothalamus, skeletal muscle and pancreatic islets, as they were based on the same cohorts (GTEx and TIGER).

### Statistics and reproducibility

All analyses were conducted using publicly available summary statistics. MR relies on genetic variants as instrumental variables, which are randomly allocated at conception and thus independent of most environmental and lifestyle confounders. No statistical method was used to predetermine sample size, but power estimations were conducted. No data were excluded from the analyses, and the investigators were not blinded to allocation during experiments or outcome assessment.

### Reporting summary

Further information on research design is available in the Nature Portfolio Reporting Summary linked to this article.

## Supplementary information

Supplementary Information Supplementary Figs. 1–3 and list of T2DGGI members.

Reporting Summary

Supplementary Tables 1–12 Supplementary Table 1: Summary of the QTL data used to select IVs for the MR analyses, including genetic ancestry group, sample size, tissue, corresponding publication and *cis*-window definition. Supplementary Table 2: Results of the blood eQTL MR analysis in the meta-analysis across ancestries and in all genetic ancestry groups assessed (EUR, AFR, AMR). For the meta-analysis results, the number of genetic ancestry groups entering the meta-analysis and the *I*^2^ value are provided. For the single-ancestry MR analyses, the number of IVs, the selected IVs, the MR method used and the PPH4 value from PWCoCo are indicated. The column ‘IVs’ reports the variants selected after our clumping approach, while the column ‘nsnp’ corresponds to the number of IVs kept after the mr_keep filtering from TwoSampleMR, explaining potential discrepancies between the two columns. Supplementary Table 3: Results of the blood pQTL MR analysis in the meta-analysis across ancestries and in all genetic ancestry groups assessed (EUR, AFR, EAS). For the meta-analysis results, the number of genetic ancestry groups entering the meta-analysis and the *I*^2^ value are provided. For the single-ancestry MR analyses, the number of IVs, the selected IVs, the MR method used and the PPH4 value from PWCoCo are indicated. The column ‘IVs’ reports the variants selected after our clumping approach, while the column ‘nsnp’ corresponds to the number of IVs kept after the mr_keep filtering from TwoSampleMR, explaining potential discrepancies between the two columns. Supplementary Table 4: Results of the blood eQTL MR analysis in the replication cohorts. Only the genes with significant causal effects in the discovery cohort are reported. For comparison purposes, the results from the blood eQTL discovery MR analyses in the corresponding genetic ancestry group are also reported. Supplementary Table 5: Results of the blood pQTL MR analysis in the replication cohorts. Only the proteins with significant causal effects in the discovery cohort are reported. For comparison purposes, the results from the blood pQTL discovery MR analyses in the corresponding genetic ancestry group are also reported. Supplementary Table 6: Number of genes and proteins with significant causal effects in the discovery MR analyses in each genetic ancestry group. The number of genes and proteins from this set tested for replication and with replicated causal effects are also reported. Supplementary Table 7: Results of the eQTL MR analysis in the seven T2D-relevant tissues (only in EUR owing to data availability). The number of IVs, the selected IVs, the MR method used, the PPH4 value from PWCoCo and the median TPM from GTEx are indicated. The column ‘IVs’ reports the variants selected after our clumping approach, while the column ‘nsnp’ corresponds to the number of IVs kept after the mr_keep filtering from TwoSampleMR, explaining potential discrepancies between the two columns. Supplementary Table 8: Chi-squared test comparing the overlap between genes with significant causal effects from the eQTL and pQTL MR analyses among the tested proteins and the proteins with significant causal effects. The test has been performed for each tissue. Supplementary Table 9: Eight curated sets of diabetes-mellitus-related genes. Gene sets were derived from multiple sources of evidence, including genetic association studies, functional genomics and prior literature. Detailed descriptions of each source and curation criteria are provided in the Methods. Supplementary Table 10: Percentage of genes tested, with significant causal estimate, and with causal effect in our causal inference analysis for each of the eight compiled DM-related sets. The percentage of tested genes corresponds to genes which were tested in any of the MR analyses, regardless of the ancestry and tissue. The significant genes correspond to genes with a *q* value lower than 5% in the corresponding MR (IVW or Wald ratio). The genes with causal effects correspond to genes with a *q* value lower than 5%, concordance in sensitivity analyses, and colocalization evidence. Supplementary Table 11: Comparison of causal effects with T2D mechanistic clusters derived in previous work^1^. For each tissue–cluster pair, a contingency table contrasting cluster membership with evidence of causal effect was constructed, and the corresponding OR was derived. The *P* value from Fisher’s exact test is reported. Supplementary Table 12: Number of genes with significant causal effects using different criteria detected in the EUR discovery cohort (eQTLGen) and tested and replicated in the EUR replication cohort (GENOA). The final column corresponds to the criteria used in our study and distinguishes the genes with only one or more than one IV. The causal effect of a gene is considered as replicated if it has a *q* value lower than 5% among the genes tested for replication with a consistent direction of effect with the discovery analysis.

## Source data

Source Data Fig. 2 Statistical source data: results from MR analyses.

Source Data Fig. 3 Statistical source data: MAF in EUR and non-EUR.

Source Data Fig. 4 Statistical source data: results from MR analyses for genes and proteins significant in any MR analysis.

Source Data Fig. 5 Statistical source data: overlap of causal genes between tissues and associated heterogeneity.

Source Data Fig. 6 Statistical source data: number of DM-related genes tested, significant and causal from MR analyses.

Source Data Fig. 7 Statistical source data: results from MR analyses for CPXM1 and HIBCH.

Source Data Extended Data Fig. 1 Statistical source data: MDE per ancestry for all genes and proteins tested.

Source Data Extended Data Fig. 2 Statistical source data: results from MR analyses for PTGES2 and TOLLIP-AS1.

Source Data Extended Data Fig. 4 Statistical source data: results from MR analyses for genes and proteins significant in the meta-analysis.

Source Data Extended Data Fig. 5 Statistical source data: results from MR analyses in T2D-relevant tissues.

Source Data Extended Data Fig. 6 Statistical source data: *z*-scores from eQTL MR analyses and overlap between tissues.

Source Data Extended Data Fig. 7 Statistical source data: overlap between tissue of causal effects and T2D cluster.

Source Data Extended Data Fig. 8 Statistical source data: comparison of genes described in Mandla et al.^23^ and in this study.

## Data Availability

All contributing cohorts have ethical approval from their institutional ethics review boards. All data used in the study are publicly available, with reference to the corresponding studies summarized in Supplementary Table 1. T2DGGI GWAS meta-analysis, https://diagram-consortium.org/downloads.html; eQTL eQTLGen, https://molgenis26.gcc.rug.nl/downloads/eqtlgen/cis-eqtl/2019-12-11-cis-eQTLsFDR0.05-ProbeLevel-CohortInfoRemoved-BonferroniAdded.txt.gz; eQTL GENOA AA and EA, http://www.xzlab.org/data/AA_summary_statistics.txt.gz and http://www.xzlab.org/data/EA_summary_statistics.txt.gz; eQTL GALAII, https://zenodo.org/records/7735723/files/MX.cis-eQTL.tar.gz?download=1; eQTL SAGE, https://zenodo.org/records/7735723/files/AA.cis-eQTL.tar.gz?download=1; pQTL deCODE, https://www.decode.com/summarydata/; pQTL ARIC, http://nilanjanchatterjeelab.org/pwas; pQTL Nagahama, https://www.hgvd.genome.med.kyoto-u.ac.jp/repository/HGV0000026.html; pQTL AASK, https://www.ebi.ac.uk/gwas/publications/35870639; pQTL UKB: ST9 from https://www.nature.com/articles/s41586-023-06592-6; eQTL GTEx, https://www.gtexportal.org/home/downloads/adult-gtex/qtl; and eQTL TIGER, https://tiger.bsc.es/downloads. Numerical source data for the figures and extended data figures are included with the paper. Source data are provided with this paper.

## Code availability

The code used to perform all single-ancestry MR analyses and the MR meta-analysis across genetic ancestry groups is available at https://github.com/Ozvan/OmicsMR.
