# Supplementary material for: Unravelling the molecular mechanisms causal to type 2 diabetes across global populations and disease-relevant tissues
Source: Nat Metab. 2026 Jan 27;8(2):506–20. doi: 10.1038/s42255-025-01444-1 (PMC12945685; doi:10.1038/s42255-025-01444-1)
Supplement: Supplementary file 2 — Reporting Summary [file 42255_2025_1444_MOESM2_ESM.pdf]

nature portfolio

Corresponding author(s): Ozvan Bocher (ozvan.bocher@univ-brest.fr
Eleftheria Zeggini
(eleftheria.zeggini@helmholtz-munich.de)

Last updated by author(s): Dec 4, 2025

Reporting Summary

Nature Portfolio wishes to improve the reproducibility of the work that we publish. This form provides structure for consistency and transparency in reporting. For further information on Nature Portfolio policies, see our Editorial Policies and the Editorial Policy Checklist.

Statistics

For all statistical analyses, confirm that the following items are present in the figure legend, table legend, main text, or Methods section.

n/a	Confirmed	
☐	☒	The exact sample size (n) for each experimental group/condition, given as a discrete number and unit of measurement
☐	☒	A statement on whether measurements were taken from distinct samples or whether the same sample was measured repeatedly
☐	☒	The statistical test(s) used AND whether they are one- or two-sided<br>*Only common tests should be described solely by name; describe more complex techniques in the Methods section.*
☐	☒	A description of all covariates tested
☐	☒	A description of any assumptions or corrections, such as tests of normality and adjustment for multiple comparisons
☐	☒	A full description of the statistical parameters including central tendency (e.g. means) or other basic estimates (e.g. regression coefficient) AND variation (e.g. standard deviation) or associated estimates of uncertainty (e.g. confidence intervals)
☐	☒	For null hypothesis testing, the test statistic (e.g. F, t, r) with confidence intervals, effect sizes, degrees of freedom and P value noted<br>*Give P values as exact values whenever suitable.*
☒	☐	For Bayesian analysis, information on the choice of priors and Markov chain Monte Carlo settings
☐	☒	For hierarchical and complex designs, identification of the appropriate level for tests and full reporting of outcomes
☐	☒	Estimates of effect sizes (e.g. Cohen's d, Pearson's r), indicating how they were calculated

Our web collection on statistics for biologists contains articles on many of the points above.

Software and code

Policy information about availability of computer code

Data collection	We performed two-sample Mendelian randomization analyses using only publicly available data, for which information is summarized in Supplemental Table 1.
Data analysis	All analyses were performed using the publicly available packages, which are cited throughout the manuscript with the corresponding version.<br>We provide the code used to perform the analyses at https://github.com/Ozvan/OmicsMR.<br>The following tools and packages were used:<br>PLINK v1.9: https://www.cog-genomics.org/plink/<br>PWCoCo: https://github.com/jwr-git/pwcoco<br>R software v4.3.3: https://cran.r-project.org/bin/windows/base/old/4.3.3/<br>R package ieugwasr v.1.0.2: https://mrcieu.github.io/ieugwasr/news/index.html<br>R package TwoSampleMR v.0.5.9: https://mrcieu.github.io/TwoSampleMR/index.html<br>R package metafor v.4.6: https://cran.r-project.org/web/packages/metafor/index.html

For manuscripts utilizing custom algorithms or software that are central to the research but not yet described in published literature, software must be made available to editors and reviewers. We strongly encourage code deposition in a community repository (e.g. GitHub). See the Nature Portfolio guidelines for submitting code & software for further information.

Data

Policy information about availability of data

All manuscripts must include a data availability statement. This statement should provide the following information, where applicable:

- Accession codes, unique identifiers, or web links for publicly available datasets
- A description of any restrictions on data availability
- For clinical datasets or third party data, please ensure that the statement adheres to our policy

All contributing cohorts have ethical approval from their institutional ethics review boards. All data used in the study are publicly available with reference to the corresponding studies summarized in Supplemental Table 1.
T2DGGI GWAS meta-analysis: https://diagram-consortium.org/downloads.html
eQTL eQTLGen: https://molgenis26.gcc.rug.nl/downloads/eqtlgen/cis-eqtl/2019-12-11-cis-eQTLsFDR0.05-ProbeLevel-CohortInfoRemoved-BonferroniAdded.txt.gz
eQTL GENOA AA and EA: http://www.xzlab.org/data/AA_summary_statistics.txt.gz ; http://www.xzlab.org/data/EA_summary_statistics.txt.gz
eQTL GALAII and SAGE: https://zenodo.org/records/7735723/files/
pQTL deCODE: https://www.decode.com/summarydata/
pQTL ARIC: http://nilanjanchatterjeelab.org/pwas
pQTL Kyoto Nagahama: https://www.hgvd.genome.med.kyoto-u.ac.jp/repository/HGV0000026.html
pQTL AASK: https://www.ebi.ac.uk/gwas/publications/35870639
pQTL UKB: ST9 from https://www.nature.com/articles/s41586-023-06592-6
eQTL GTEx: https://www.gtexportal.org/home/downloads/adult-gtex/qtl
eQTL TIGER: https://tiger.bsc.es/downloads

Research involving human participants, their data, or biological material

Policy information about studies with human participants or human data. See also policy information about sex, gender (identity/presentation), and sexual orientation and race, ethnicity and racism.

Reporting on sex and gender	We used summary statistics publicly available for the QTL and T2D GWAS datasets and did not have access to the individual data. Sex was used as a covariate in the regression models used to generate the different summary statistics.
Reporting on race, ethnicity, or other socially relevant groupings	One of the main aim of the paper is to evaluate the ancestry-related heterogeneity of causal molecular effects to T2D. We performed single-ancestry MR analyses using exposure and outcome data from matched genetic ancestry groups, and then meta-analyzed the results across ancestry groups. The ancestries considered corresponds to the ones reported in each of the cohort used in our study. Although genetic ancestry exists on a continuous spectrum, we refer to continental ancestry groupings defined by the 1000 Genomes Project Phase 3, which include African (AFR), East Asian (EAS), European (EUR), South Asian (SAS), and Admixed American (AMR) populations.
Population characteristics	We only used summary statistics and did not have access to individual data. We describe the sample size of the QTL studies as well as the number of cases and controls in each ancestry group in the T2D GWAS meta-analysis in Supplemental Table 1 and in Figure 1. Readers can refer to publications corresponding to each dataset for more information.
Recruitment	No participants were recruited in this study as we used only summary statistics publicly available.
Ethics oversight	Ethical approval was not directly obtained from this study which is solely based on summary statistics. All contributing cohorts (described in Supplemental Table 1) obtained ethical approval from their institutional ethics review boards.

Note that full information on the approval of the study protocol must also be provided in the manuscript.

Field-specific reporting

Please select the one below that is the best fit for your research. If you are not sure, read the appropriate sections before making your selection.

☒ Life sciences ☐ Behavioural & social sciences ☐ Ecological, evolutionary & environmental sciences

For a reference copy of the document with all sections, see nature.com/documents/nr-reporting-summary-flat.pdf

Life sciences study design

All studies must disclose on these points even when the disclosure is negative.

Sample size	No sample size calculation was performed within this study as only publicly summary statistics were used. Cohorts were chosen based on availability in the different genetic ancestry groups. When multiple cohorts were available for a given genetic ancestry group, we used the cohort with the largest sample size for discovery purposes, and smaller cohorts for replication to maximize statistical power. The sample sizes of each QTL cohort used as exposure is described in Supplemental Table 1, and ranges from a few hundreds to several thousands of individuals. We acknowledge that differences in sample sizes result in differences in statistical power of our Mendelian randomization analysis, a point which is emphasized in the paper.
Data exclusions	No data was excluded in the analyses, summary statistics downloaded from the diffreent sources were directly used.

Replication	Our work is solely based on computational approaches and data analysis. However, we acknowledge that potential biases can arise in Mendelian randomization analyses. To strenghten the confidence in our results, we performed sensitivity analyses and colocalization. Additionally, we performed replication, wherever possible, in independent cohorts from matched ancestry groups. We describe a total of 79 genes and/or proteins which are replicated with concordant direction of effect, and highlighted throughout the manuscript in the figures and results. Due to the lack of available data, replication could not be conducted in the seven T2D-relevant tissues.
Randomization	This is not relevant to our study.
Blinding	This is not relevant to our study.

Reporting for specific materials, systems and methods

We require information from authors about some types of materials, experimental systems and methods used in many studies. Here, indicate whether each material, system or method listed is relevant to your study. If you are not sure if a list item applies to your research, read the appropriate section before selecting a response.

Materials & experimental systems

n/a	Involved in the study
☒	☐ Antibodies
☒	☐ Eukaryotic cell lines
☒	☐ Palaeontology and archaeology
☒	☐ Animals and other organisms
☒	☐ Clinical data
☒	☐ Dual use research of concern
☒	☐ Plants

Methods

n/a	Involved in the study
☒	☐ ChIP-seq
☒	☐ Flow cytometry
☒	☐ MRI-based neuroimaging

Plants

Seed stocks	*Report on the source of all seed stocks or other plant material used. If applicable, state the seed stock centre and catalogue number. If plant specimens were collected from the field, describe the collection location, date and sampling procedures.*
Novel plant genotypes	*Describe the methods by which all novel plant genotypes were produced. This includes those generated by transgenic approaches, gene editing, chemical/radiation-based mutagenesis and hybridization. For transgenic lines, describe the transformation method, the number of independent lines analyzed and the generation upon which experiments were performed. For gene-edited lines, describe the editor used, the endogenous sequence targeted for editing, the targeting guide RNA sequence (if applicable) and how the editor was applied.*
Authentication	*Describe any authentication procedures for each seed stock used or novel genotype generated. Describe any experiments used to assess the effect of a mutation and, where applicable, how potential secondary effects (e.g. second site T-DNA insertions, mosiacism, off-target gene editing) were examined.*
